# Predisposing and precipitating factors for delirium in community-dwelling older adults admitted to hospital with this condition: A prospective case series

**DOI:** 10.1371/journal.pone.0193034

**Published:** 2018-02-23

**Authors:** Emmanuelle Magny, Hélène Le Petitcorps, Maria Pociumban, Zineb Bouksani-Kacher, Éric Pautas, Joël Belmin, Sylvie Bastuji-Garin, Carmelo Lafuente-Lafuente

**Affiliations:** 1 Service de Gériatrie à orientation Cardiologique et Neurologique, Hôpital Charles Foix, Hôpitaux universitaires Pitie-Salpêtrière-Charles Foix, APHP, Paris, France; 2 Service de Gériatrie aiguë, Hôpital Charles Foix, Hôpitaux universitaires Pitie-Salpêtrière-Charles Foix, APHP, Paris, France; 3 Plateforme de Recherche Clinique en Gériatrie, Hôpital Charles Foix, Hôpitaux universitaires Pitie-Salpêtrière-Charles Foix, APHP, Paris, France; 4 DHU FAST, Sorbonne Universités, Université Paris 6 Pierre et Marie Curie (UPMC), Paris, France; 5 Université Paris Est Créteil (UPEC), IMRB, A-TVB DHU, CEpiA EA 4393, (Clinical Epidemiology and Ageing Unit, APHP, Hôpital Henri Mondor, Service de Santé Publique, Créteil, France; University of Brescia, ITALY

## Abstract

**Background:**

Factors associated with delirium among community-dwelling older adults have been poorly studied. Our aim was to describe the prevalence of predisposing and precipitating factors for delirium among patients admitted for delirium and to assess whether these factors were appropriately recognized at the first patient assessment at hospital.

**Methods:**

Consecutive community-dwelling individuals admitted to three geriatric acute care units with a confirmed initial diagnosis of delirium were prospectively included. An independent investigator recorded, using a predefined form, any acute medical condition considered by the attending geriatrician to be a precipitating factor, at the first patient assessment and at the end of his stay in acute care.

**Results:**

A total of 208 patients were included, 80.0% had a pre-existing cognitive or neurological disorder, or both. The most frequent precipitating factor found were infections (49.0% of all patients, mainly lung and urinary tract infections), followed by drugs (30.8%), dehydration (26.4%) and electrolytic disturbances (18.7%, mostly hyponatremia). 91% of patients had a cerebral imagery, but acute neurological conditions were found in only 18.3%. Fewer precipitating factors were found at first than at final assessment (1.4 (95%CI 1.3–1.6) versus 1.9 (95%CI 1.8–2.0) respectively, p<0.001). This difference was significant for all main categories of precipitating factors.

**Conclusions:**

Infections, followed by drugs and hydro-electrolytic disorders seem to be the most frequent precipitating factors for delirium in community-dwelling elderly individuals. Early diagnostic and management of precipitating factors in these patients should be improved, as a significant number of them are missed at the initial assessment.

## Introduction

Delirium, or acute confusional state, is a clinical syndrome characterized by disturbances in attention, awareness and cognition (such as in memory, orientation, language, and perception) that develop over a short period of time, are not better explained by another pre-existing neurocognitive disorder and are a direct consequence of another medical condition, substance intoxication or withdrawal, or exposure to a toxin[**1**]. Patients affected are more often old people [**1**]. Delirium is typically a multifactorial condition: it usually involves a predisposing, pre-existing factor–often a cognitive disorder–and one or several acute, superimposed, organic disease(s) that directly precipitate the delirium [**2**, **3**]. Its physiopathology is not completely understood but it involves a disruption of large-scale neuronal networks [**1, 4**].

Even if delirium is usually temporary and reversible, patients who developed delirium had longer hospital stays, increased short-term mortality and a much higher frequency of dementia diagnosis at follow-up, compared with similar patients without delirium [**4 – 7**]. There is accumulating evidence that supports the hypothesis that delirium itself contributes to, or mediates, permanent cognitive impairment [**4**, **5**].

Delirium occurring in in-hospital patients has been extensively studied. Large case series and case-control studies have reported the most important predisposing and precipitating factors [**1, 3, 8, 9**]. Precipitating factors vary depending on the setting. In medical wards, polypharmacy, use of psychoactive drugs, physical restraints and renal failure were leading factors [**1**, **9**].

Delirium is also a frequent condition in community-dwelling elderly people. In this setting, the prevalence of delirium has been reported to be 1 to 2% [**1, 10**], but its prevalence increases markedly with age and may reach as much as 21% among individuals aged 90 or over [**11**]. The occurrence of delirium usually brings the patient to emergency care and hospital admission. Thus, delirium is present in 8–17% of elderly people presenting to emergency department [**1**], and it exists at admission in 10%– 31% of elderly patients acutely admitted to hospital [**5**]. Delirium in the community has been, however, less studied. Only two studies have reported prevalence rates of predisposing and precipitating factors of delirium in community-dwelling old patients and their results are not consistent: infections, metabolic disturbances and adverse drug effects were the most frequent triggers of delirium in one study [**12]**, while in the other they were metabolic disturbances and acute cardiovascular diseases [**13**].

Early recognition of delirium, followed by rapid diagnosis and treatment of all existing precipitating factors is essential to provide patients with the best chances of recovery [**1**, **6, 7**]. To this end, it is important to know precisely which the most common precipitating factors are. Therefore, we conducted a prospective study with the main objective of estimating the frequency of the different predisposing and precipitating factors for delirium occurring in community-dwelling older patients admitted to hospital with this diagnostic. A secondary objective was to assess whether precipitating factors were correctly recognized at the first clinical assessment at hospital of these patients.

## Methods

### Study design

A prospective, consecutive case series.

### Ethics

All included patients and their families received written information about the use of their personal medical data for the study and could refuse at any moment that their data were included. Because patients were, by definition, delirious at inclusion and because it is not required under French law for purely observational studies, we did not ask for written consent. Data was anonymized. The *Comité consultatif sur le traitement de l’information en matière de recherche dans le domaine de la santé* (CCTIRS), from the French *Commission Nationale Informatique et Libertés (CNIL)* authorized the use of medical data for this study (Approval number: 1806824v0). The study was conducted in full accordance with the Declaration of Helsinki.

### Setting

Three geriatric acute care units of the Charles Foix hospital, a geriatric center part of a large teaching hospital group, the Hôpitaux Universitaires Pitié-Salpêtrière-Charles Foix. Charles Foix hospital covers a densely urbanized geographic area south of Paris, France, and admits patients for acute care from several hospital emergency departments and directly from home or nursing homes, when sent by a general practitioner. The three participant units accumulate a total of 102 acute care beds.

### Patients

All patients admitted from November 2014 to April 2016 to one of the participating geriatric acute care units with an initial diagnosis of delirium were prospectively included. Delirium could be either the main reason for referral to hospital or a secondary diagnosis, but it had to be present at the first patient examination at hospital. Diagnosis of delirium was systematically confirmed by one of us using the Confusion Assessment Method (CAM) [**14**]. Only patients admitted from home, nursing home or retirement apartments (directly or through the emergency department) were included. Patients referred by other hospital department or after having been for more than 48 hours at the emergency department were excluded.

### Measurements

Data were prospectively collected by one of us (EM, MP, HL, ZBK, CLL), using a standardized form, from the patient’s medical record and by questioning his attending doctor. The variables collected are listed in **Tables 1** and **2**. Precipitating factors were recorded twice, at the first medical evaluation and at the last assessment performed in acute care, once all clinical and complementary examinations were completed. Only those precipitating factors that were established to be already present at patients’ admission were considered (e.g. symptoms of infection present at arrival or a positive bacteriological sample taken the first day, but not afterwards).

**Table 1:** Characteristics of included patients and potential predisposing factors.

Variables	No. (%) n = 208
Age, *years* (mean, 95%CI)	86 (85–87)
Women	143 (68.7)
Living:	
Home	180 (86.5)
Nursing home	20 (9.6)
Retirement apartments (independent or assisted living)	8 (3.8)
Sensory deficits	
Visual: Mild	11 (5.3)
Moderate	57 (27.4)
Severe	14 (6.7)
Hearing: Mild	6 (2.9)
Moderate	25 (12.0)
Severe	16 (7.7)
History of cognitive disorder:	154 (74.0)
Dementia:	109 (52.4)
Type unspecified	74 (35.6)
Alzheimer’s disease	25 (12.0)
Mixed Alzheimer’s & vascular	5 (2.4)
Lewy body dementia	4 (1.9)
Vascular dementia	1 (0.5)
Long-lasting cognitive impairment, undiagnosed	45 (21.6)
History of neurological disease:	50 (24.0)
Cerebrovascular disease	35 (16.8)
Parkinson disease	35 (16.8)
Epilepsy	7 (3.4)
Cerebral tumor, non-malignant	1 (0.5)
History of psychiatric disease:	63 (30.3)
Depression	48 (23.1)
Psychosis	6 (2.9)
Anxiety disorders	3 (1.4)
Unspecified	6 (2.9)
Outcomes at the end of hospital stay (*n = 204*):	
Returning home	134 (65.7)
Nursing home / retirements apartments	46 (22.6)
Death	24 (11.8)
Transferred to subacute / specialized care before leaving hospital	99 (48.5)
Length of hospital stay, *days* (median (IQR)), (*n = 204*):	
In acute care	15 (11–19)
Total stay at hospital	23 (14–45)

95%CI = 95% Confidence interval; IQR = Interquartile range; SD = Standard deviation.

**Table 2:** Acute conditions found and deemed precipitating factors for delirium in included patients.

Condition	At first assessment No. (%) n = 208	At final assessment No. (%) n = 208	P value*
Infection	76 (36.5)	103 (49.5)	< 0.001
Lung	34 (16.3)	46 (22.1)	
Pneumonia	28 (13.5)	43 (20.7)	
Acute bronchitis (1 COPD)	6 (2.9)	3 (1.4)	
Urinary	23 (11.1)	32 (15.4)	
Fever or SIRS, unclear etiology	13 (6.2)	11 (5.3)	
Skin	4 (1.9)	6 (2.9)	
Intra-abdominal	1 (0.5)	4 (1.9)	
Others (grippe, osteomyelitis, sepsis)	1 (0.5)	4 (1.9)	
Water and electrolyte disorders	76 (36.5)	95 (45.7)	< 0.001
Volume depletion, normal natremia	47 (22.6)	55 (26.4)	
Hyponatremia	20 (9.6)	26 (12.5)	
Hypernatremia	8 (3.8)	10 (4.8)	
Hypercalcemia	1 (0.5)	3 (1.4)	
Drugs	45 (21.6)	64 (30.8)	< 0.001
Psychotropic drugs	39 (18.7)	49 (23.6)	
Benzodiazepines	18 (8.7)	24 (11.5)	
Antidepressants	4 (1.9)	8 (3.8)	
Antipsychotics	7 (3.4)	7 (3.4)	
Anti-Parkinson	4 (1.9)	6 (2.9)	
Sedating antihistamines	4 (1.9)	6 (2.9)	
Anti-Alzheimer (memantine, anticholinesterases)	3 (1.4)	3 (1.4)	
Others (lithium, levetiracetam)	2 (1.0)	2 (1.0)	
Opioids	6 (2.9)	9 (4.3)	
Fluoroquinolones	0	3 (1.4)	
Others (oral anti-diabetic, NSAID, oral corticoid, furosemide)	1 (0.5)	5 (2.4)	
Acute neurological condition	28 (13.5)	38 (18.3)	0.01
Epilepsy	10 (4.8)	14 (6.7)	
Subdural hematoma	11 (5.3)	11 (5.3)	
Stroke	6 (2.9)	11 (5.3)	
Ischemic	5 (2.4)	9 (4.3)	
Intra-cerebral hemorrhage	1 (0.5)	2 (1.0)	
Head trauma, no intra-cranial hemorrhage	6 (2.9)	6 (2.9)	
Others acute diseases	19 (9.1)	29 (13.9)	0.002
Heart failure	8 (3.8)	13 (6.2)	
Hypoglycemia	4 (1.9)	4 (1.9)	
Alcohol abuse	2 (1.0)	3 (1.4)	
Pulmonary embolism	2 (1.0)	2 (1.0)	
Fall-related fracture	2 (1.0)	2 (1.0)	
Cancer, disseminated	1 (0.5)	2 (1.0)	
Other (hyperthyroidism, sleep apnea, temporal arteritis)	0	3 (1.4)	
Others conditions found, uncertain role **			
Urinary retention	16 (7.7)	26 (12.5)	
Pain	18 (8.7)	23 (11.1)	
Fecal impaction †	13 (6.2)	14 (6.7)	
Arrhythmia	1 (0.5)	1 (0.5)	
Physical aggression	0	1 (0.5)	
Recent admission to nursing home	0	1 (0.5)	

COPD = Chronic obstructive pulmonary disease; NSAID = Non-steroidal inflammatory drug; SIRS = Systemic inflammatory response syndrome;

* McNemar test for proportions in two paired samples

** They were considered by the attending physician as a probable precipitating factor but are not clearly recognized as such by all experts (see text).

**†** Only one patient with this diagnosis had clinical signs of intestinal occlusion

We considered as potential predisposing factors any pre-existing cognitive disorder or neurological disease. As for precipitating factors, we recorded any acute organic medical condition deemed by the patient’s attending doctor–always a qualified geriatrician–to be a trigger for delirium. Concerning drugs, only drugs recently prescribed or whose dosing was recently modified were considered. Drugs chronically prescribed with no modification were not retained. We grouped in a particular category some conditions, such as pain, urinary retention or faecal impaction that some specialists consider may precipitate delirium but that are not recognized by all experts as a cause of delirium or that can be a consequence, rather than a cause of delirium [**1, 5, 9, 15–17**].

### Statistics

We described the prevalence of predisposing and precipitating factors using numbers (proportions). Mean and 95% confidence interval (95%CI) were employed to describe normally distributed data and median and interquartile range (IQR) for data that were not normally distributed. To assess whether the findings at the first and the final assessment differed, we compared the number of precipitating factors using a paired Student test, and the prevalence of each of them, using a paired McNemar test. All tests were two-sided and the threshold for significance was set at 0.05. STATA software (version 13.1 SE, StataCorp LP, Texas, USA) was employed for data management and statistics.

We followed the STROBE statement for improving the reporting of observational studies while writing this manuscript [**18**]. A completed STROBE checklist is available from the authors upon request.

## Results

**Fig 1:**
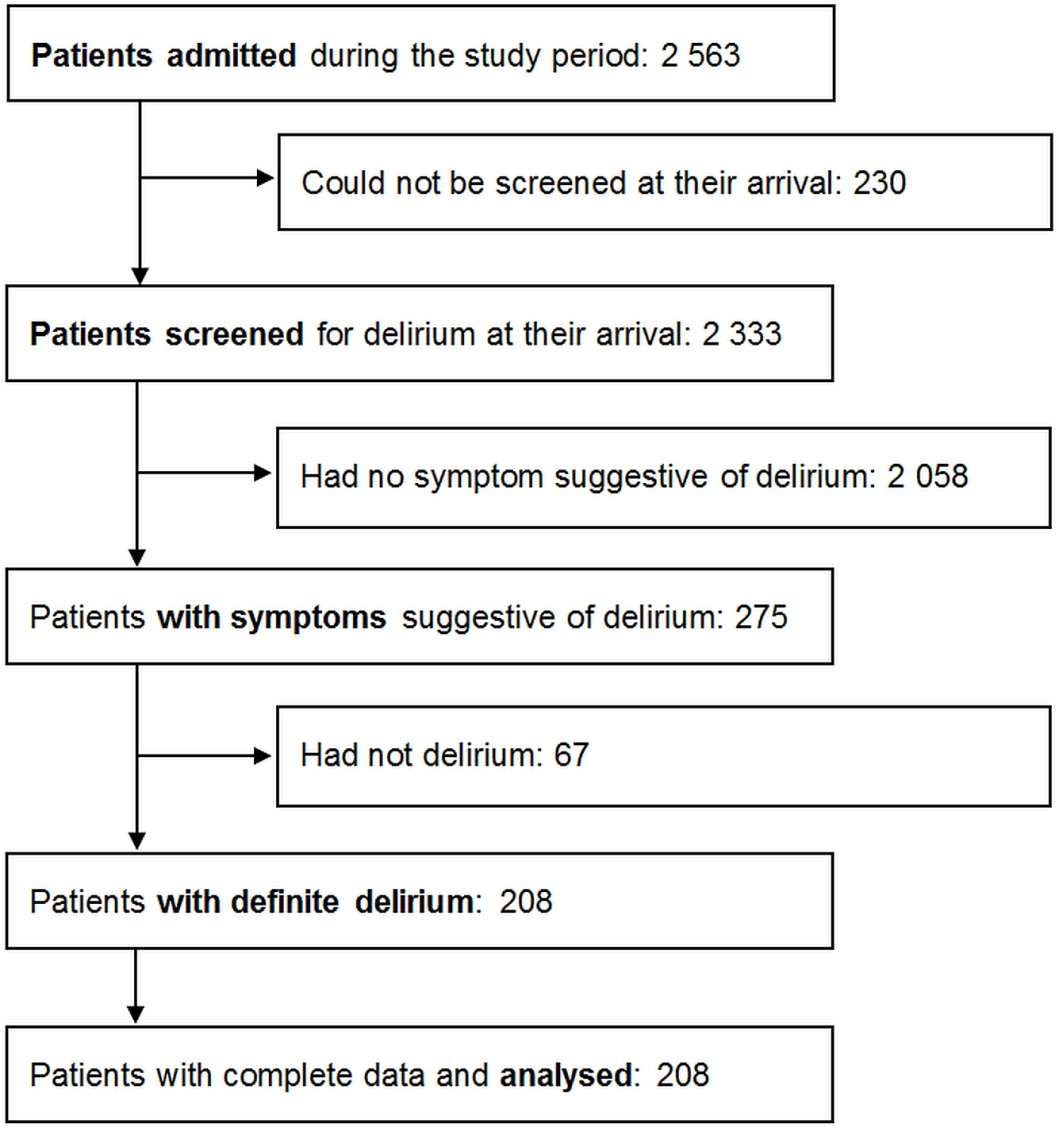
Inclusion of patients.

Out of 2563 patients admitted during the study period, 208 patients had confirmed delirium and were included (**Fig 1**). Characteristics of included patients are shown in **Table 1**.

Mean age was 86 years old (age range, 68 to 103 years) and 68.7% were women. Most patients were living at home (86.5%) and half of them lived alone. Confusion, altered mental status or abnormal behaviour were the main reason for referral to hospital in 155 patients (76.0%); in the remaining patients, delirium was an associated diagnostic.

Median stay at hospital was 23 days (IQR 14 to 45 days). In-hospital mortality was 11.6%. Only 65.7% of all patients were able to return back home after their hospitalization.

### Probable predisposing factors

The majority of patients (74.0%) had a pre-existing cognitive disorder: 52.4% had a previous diagnosis of dementia of various types; another 21.6% had a history of pre-existing chronic cognitive impairment, noted by relatives or caregivers, which had not been further studied (**Table 1**).

Fifty patients (24.0%) had a history of diverse neurological diseases. Cerebrovascular disease and Parkinson’s were the most frequent ones (**Table 1**). Overall, 80.0% of patients had a pre-existing cognitive or neurological disorder, or both.

Finally, 30.3% of patients had a psychiatric disorder, mostly depression.

### Potential precipitating factors

Details on precipitating factors are displayed in **Table 2**. The most frequent conditions considered, at patients’ final assessment, as having been precipitating factors for delirium were: 1) infections (49.5% of patients), 2) hydro-electrolytic disorders (45.7%) and, 3) adverse drug reactions (30.8%). Overall, 172 patients (82.7%) had one or several acute conditions belonging to these three categories.

The leading infections were respiratory (22.1% of all patients) and urinary tract infections (15.4%). No case of meningitis or meningoencephalitis was diagnosed. The most frequent hydro-electrolytic disturbances were dehydration (26.4% of patients) and hyponatremia (12.5%). Between the drugs incriminated in triggering delirium in this series, psychotropic drugs were by far the most frequent (23.6% of all patients), specially benzodiazepines. 91% of patients had a cerebral imagery (CT and/or RMI), but acute neurological conditions of any kind were found in only 18.3% of patients.

Finally, certain conditions, such as pain, urinary retention or faecal impaction that are not universally recognized as a cause of delirium, or that can be a consequence of delirium, were considered as precipitating factors by some attending physicians (**Table 2)**.

Patients had on average 1.9 different precipitating factors (SD = 1.0) (**Table 3**). No precipitating factor at all was found in 5.3% of patients.

**Table 3:** Number of precipitating factors found at first and final assessment.

Number	First assessment N (%)	Final assessment N (%)	P value*
0	24 (10.6)	11 (5.3)	
1	101 (48.6)	69 (33.2)	
2	55 (26.4)	68 (32.7)	
3	22 (10.6)	49 (23.6)	
4	6 (2.9)	10 (4.8)	
5	0	1 (0.5)	
Mean (95%CI)	1.4 (1.3–1.6)	1.9 (1.8–2.0)	< 0.001

95%CI = 95% Confidence Interval

* Paired Student t test

### Comparison with the initial assessment

**Fig 2:**
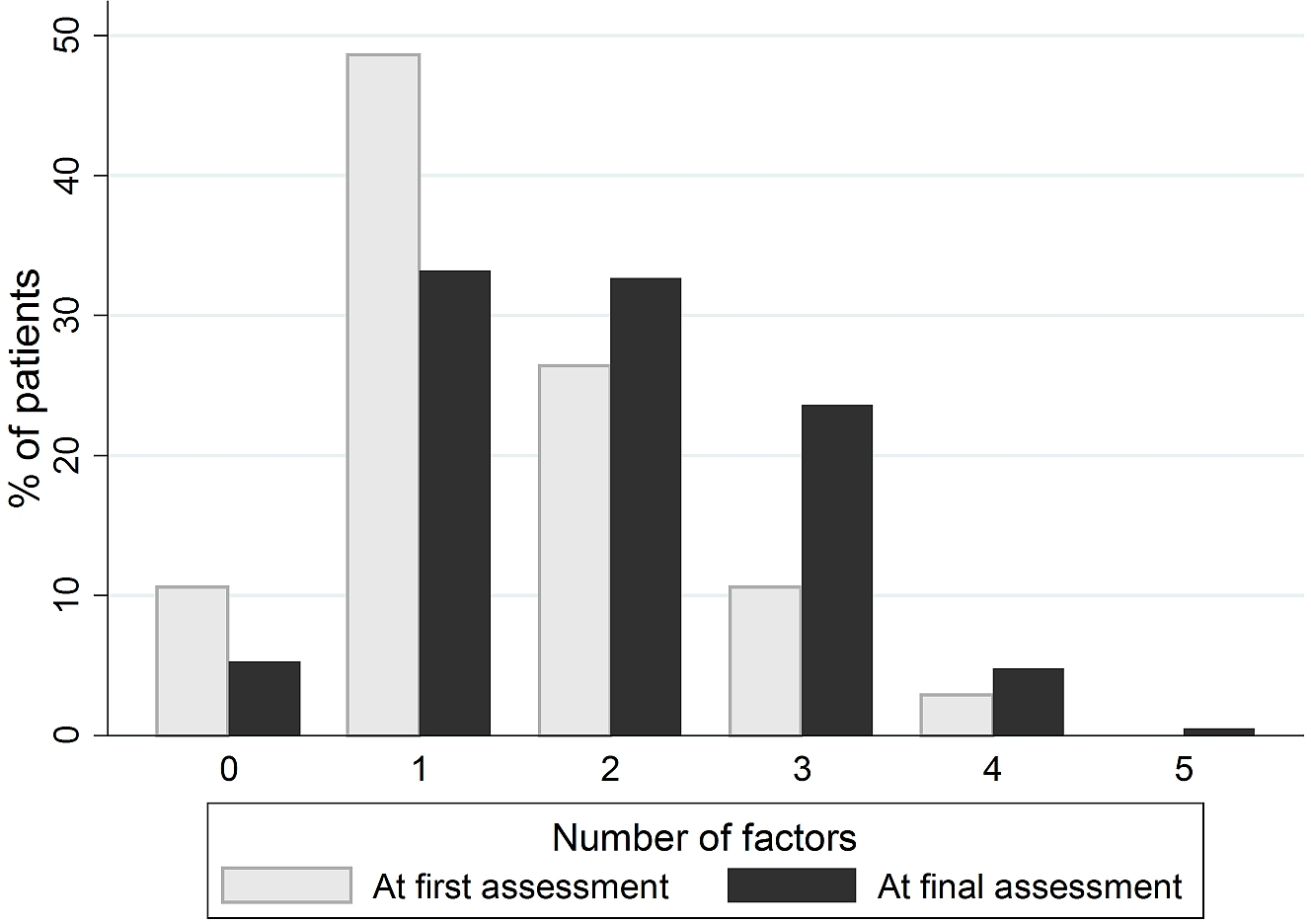
Number of precipitating factors found at first and final assessment.

Fewer precipitating factors were found at first than at final assessment (mean 1.4 (95%CI 1.3 to 1.6) *versus* 1.9 (95%CI 1.8 to 2.0) respectively, p < 0.001) (**Table 3** and **Fig 2**). The difference was significant for all four most frequent categories of precipitating factors: infections, hydro-electrolytic disorders, drugs and acute neurological conditions (**Table 2)**. Infections were missed at initial assessment in 12.5% of patients, hydro-electrolytic disorders in 9.2%, drugs in 9.1% and acute neurological conditions in 4.8% of patients.

## Discussion

Delirium developing in elderly in the community has been less extensively studied than delirium in hospital settings. In this work, we have prospectively recorded in a systematic way any possible predisposing and precipitating factor in 208 consecutive patients admitted to hospital after developing delirium at home. Delirium was indeed the main reason for admission in most of these patients (76.0%).

What we found in this study is that: a) a large majority of these patients had pre-existing predisposing conditions, b) infections appear as the most frequent precipitant, considerably more than in-hospital delirium, and c) a significant number of precipitating conditions are missed at the first patient assessment. Otherwise, the nature and diversity of predisposing and precipitating factors we found in community-dwelling patients are not fundamentally different from what has been described in other settings [**1 – 3, 8, 9**].

Regarding predisposing factors, the main finding in this study was the very high prevalence of pre-existing dementia or cognitive impairment, which were present in 74% of all included patients. In many cases (22% of all patients) the cognitive impairment had been noted by the patient’s relatives but had not been medically assessed. In most studies conducted in in-hospital settings, a history of dementia or cognitive impairment was found in only 30 to 50% of patients with delirium [**19**].

With respect to precipitating factors, infections, hydro-electrolytic disorders and drug adverse effects were the most frequent categories in this series. One or the other were present in 83% of patients in this study. Dehydration and electrolytic disturbances, as well as drugs–particularly psychoactive drugs, as in our study–are also frequent precipitants in all settings at hospital [**1, 2, 7 – 9, 17, 19, 20**]. What is striking in this study, compared to in-hospital studies, is the higher frequency of infections, which were present in half of all patients (49.5%), with pulmonary and urinary tract infections being the most frequent ones. By contrast, in-hospital studies, either in medical or in surgical wards, have reported a lower frequency of infections: 15 to 37% of patients with delirium [**1, 19, 21, 22**].

Only two studies on delirium in the community have been published to date. The largest of them, by *Laurila et al*, prospectively studied a series of 87 patients with delirium and obtained results very similar to ours [**12**]. They found the same three more frequent categories of precipitants. Infections were present in as much as 69% of all patients. The other study on community-dwelling patients, *by Elmstahl et al*, identified metabolic disturbances (30%) and cardiovascular diseases (16%) as the most frequent precipitating factors [**13**]. This study was, however, retrospective, and included only 56 cases of delirium diagnosed among emergency department patients.

Our results, together with those of *Laurila et al*, suggest that infections are a much more frequent precipitating factor for delirium in community-dwelling patients than in in-hospital patients. This has important clinical implications, as infections usually have a very effective treatment if they are correctly diagnosed, but otherwise they can evolve rapidly.

However, other key finding of this study is the observation that a significant number of precipitant factors, in all categories, were missed at the initial assessment of patients. This phenomenon affected specially the more frequent precipitants–infections, hydro-electrolytic disorders and drugs–though they are precisely the most easily modifiable factors. Missing precipitant factors at the first patient evaluation is not completely unexpected. Information available the first time a doctor see a patient is often incomplete. Moreover, clinical manifestations of acute illnesses are frequently atypical or non-specific in elderly patients–even more if the patient has delirium–including the clinical presentation of common infectious diseases: fever may be absent or blunted and the most frequent signs are non-specific (falls, anorexia, weakness…) [**23, 24**].

All that can explain why precipitating factors are easily missed at the first patient evaluation, but this is far from optimal. Prompt recognition and treatment of all modifiable etiologic factors is a mainstay of optimal delirium management [**17**, **18, 25**]. It has been observed that a delayed diagnosis of delirium or its precipitants is associated with increased mortality [**26**] and that every 48 hours spent with delirium is associated with an 11% increase in mortality [**6**].

Poor understanding of delirium probably also contributes to a delayed diagnostic of precipitating factors. In our experience, initial assessment of elderly patients with delirium is often focused on the search for neurologic causes (cerebral imagery was performed in 91% of patients included in our study). However, if we follow the findings of this study, we should first search for infections, dehydration, sodium abnormalities and review any medication taken by the patient.

Finally, another question is what could be done to prevent precipitating factors in vulnerable older adults in the community. Ensuring appropriate prescription of any medication would be essential, with careful consideration before introducing any new drug, especially any psychotropic drug. Influenza and, possibly, pneumococcal vaccination might reduce the number of severe respiratory infections. Last but not least, caregivers, who have a crucial role in this setting, could be teach how to promote a healthy hydration, to recognize delirium and to react rapidly to it.

The main **limitation** of our study is that we studied patients with delirium referred to hospital but did not followed a cohort of older people in the community. Consequently, it is not sure that the patients here included were representative of the general population of elderly patients developing delirium in the community. Nonetheless, most patients who develop delirium in this setting are referred to hospital, so studying those patients is a good proxy. In any case, all patients included in this study developed delirium in the community, before arriving to hospital, and they represent a different population than patients who develop delirium after admission at hospital.

Other potential limitations are that: a) there is limited evidence on the validity of the CAM (or any other tool) to detect delirium superimposed on patients with dementia, which might introduce a measurement bias [**27**]; b) given the relatively limited number of patients studied, some less frequent causes of delirium might have been missed. Other, independent, studies would be needed to confirm and expand our findings. Finally, it could be criticized the fact that we did not include a control group of similar patients without delirium. In fact, our objective was to obtain a general description of the more frequent predisposing and precipitating conditions existing in these patients, rather than determine which factors were different in patients with and without delirium.

In **conclusion**, we found in this series of patients who developed delirium in the community and were admitted to hospital, that the most frequent precipitating factor in this setting appear to be infections (mostly respiratory and urinary), followed by dehydration and electrolytic disturbances (mainly hyponatremia) and drugs (particularly psychotropic drugs). Infections, in particular, seem to be considerably more frequent in patients who develop delirium in the community than in patients at hospital. Acute neurological or cardiovascular diseases were much less frequent.

Finally, we found evidence that a significant number of precipitating factors, in all categories, are frequently missed at the first evaluation of patients with delirium. A systematic initial search for these very frequent conditions–infections, hydro-electrolytic disorders and drugs–could accelerate the diagnosis and treatment of modifiable causes of delirium. This, in turn, might shorten the duration of delirium and lead to improved final outcomes. This strategy, however, would need to be proven effective in clinical trials. Other strategy would be to develop and test effective methods to prevent delirium in patients at risk in the community.
